# Hypertension and cystatin C account for sex differences in serum homocysteine levels in acute coronary syndrome subjects with normal serum creatinine

**DOI:** 10.1186/s41043-023-00430-1

**Published:** 2023-08-16

**Authors:** Kun Shang, Xiang Ning, Jiangying Kuang, Aiying Xue, Xiao Yan, Huiqiang Chen

**Affiliations:** 1 Department of Pediatric Oncology, Shandong Cancer Hospital and Institute, Shandong First Medical University and Shandong Academy of Medical Sciences, Jiyan Road, No. 440, Jinan, Shandong China; 2 Department of Cardiology, The Second Hospital, Cheeloo College of Medicine, Shandong University, Jinan, 250012 Shandong China

**Keywords:** Sex difference, Acute coronary syndrome, Homocysteine, Cystatin C, Hypertension, Creatinine

## Abstract

**Background:**

Hyperhomocysteinemia is one of cardiovascular disease risk factors and fasting homocysteine levels are significantly elevated in male compared to female acute coronary syndrome (ACS) patients with normal renal function. However, it is not known the sex related determinants of plasma homocysteine levels in ACS subjects without renal dysfunction.

**Methods:**

A total of 165 ACS participants with normal plasma creatinine who underwent coronary angiography were included in the present study. Clinical parameters, homocysteine, fasting glucose and lipid profile, hemoglobin, white blood cell, platelets, creatinine, cystatin C, blood urea nitrogen, uric acid (UA), and albumin were measured. Multivariate linear regression analyses were used to recognize the predictive factors for homocysteine.

**Results:**

The levels of plasma homocysteine were significantly higher in men than in women (*P* < 0.0001). In males, homocysteine (log10) was positively associated with hypertension (*r* = 0.569, *P* < 0.001), creatinine (*r* = 0.367, *P* < 0.001) and cystatin C (log10) (*r* = 0.333, *P* = 0.001). In females, homocysteine (log10) was positively correlated with age (*r* = 0.307, *P* = 0.107), hypertension (*r* = 0.456, *P* < 0.001), creatinine (*r* = 0.341, *P* = 0.008), cystatin C (log10) (*r* = 0.429, *P* = 0.001) and UA (*r* = 0.569, *P* < 0.001) whereas was negatively associated with LDL-C (*r* =  − 0.298, *P* = 0.021) and ApoB (*r* =  − 0.273, *P* = 0.033). Parameters up to statistical significance in males or females were incorporated into the stepwise linear regression models. In men, hypertension (*P* < 0.001) and creatinine (*P* = 0.031) were independently related to homocysteine. Most of the variability of homocysteine levels in males were only determined by hypertension. In women, cystatin C (log10) (*P* = 0.004) and hypertension (*P* = 0.005) were independently related to homocysteine (log10). Plasma cystatin C had a higher explanatory value than hypertension in females.

**Conclusions:**

Hypertension and cystatin C could explain most of the sex differences in serum homocysteine levels in ACS subjects with normal serum creatinine. This finding suggested the importance of making different strategies in males and females to manage hyperhomocysteinemia effectively in ACS subjects without renal dysfunction.

## Introduction

Homocysteine is a sulfur-containing amino acid which is generated during methionine and cysteine metabolism. Hyperhomocysteinemia, which refers to the elevated plasma total homocysteine levels, is an independent risk factor for stable ischemic heart disease [1] as well as acute coronary syndrome (ACS) [1–3]. A growing body of research showed that hyperhomocysteinemia was related to an increased chance of major adverse cardiovascular events and all-cause mortality among ACS patients in males [4] as well as in females [2, 3]. Of note was that fasting plasma homocysteine levels were significantly elevated in adult men compared to women among ACS subjects [5], the sex difference of which has not been illustrated completely in cardiovascular diseases. Thus, exploring the gender-specific expression and the predictive factors of homocysteine in ACS participants is necessary.

The sex differences of fasting plasma homocysteine concentrations above could not be well explained by the circulating sex hormone levels since no significant relationship was found between testosterone, DHEA sulfate, and estradiol levels with fasting plasma homocysteine levels in middle-aged and elderly men [6]. It seemed that the gender-related alterations of fasting plasma homocysteine might be mainly determined by age and renal dysfunction since age and kidney function were the primary correlates of fasting plasma total homocysteine levels in non-diabetic and diabetic adults [7]. The increase of circulating homocysteine levels with age could partly be attributed to the age-related deterioration of renal function [8], raising the possibility that the sex differences of fasting plasma homocysteine concentrations in ACS might be explained by kidney dysfunction. In fact, renal function is an important determinant of serum homocysteine levels. There is a positive association between serum homocysteine and renal function parameters including creatinine and cystatin C in renal failure patients [9] or in normal renal function subjects [10]. Cystatin C levels could independently predict fasting plasma homocysteine levels among stable coronary heart disease participants with normal serum creatinine levels [11]. As to ACS subjects, however, the raised plasma homocysteine concentration had not be well explained by the subtle renal dysfunction [12]. In this regard, it is necessary to assess the sex-related determinants of plasma total homocysteine levels in ACS subjects with normal renal function. Clarifying this issue could shed some light on the cause and the management of high homocysteine levels in ACS subjects without renal dysfunction.

In the present study, we aimed to explore the sex differences in the determinants of plasma total homocysteine levels in ACS participants with normal serum creatinine.

## Patients and methods

### Patient selection

The participants were screened from inpatients with the symptoms of chest discomfort who underwent coronary angiography at the Second Hospital of Shandong University in China from Sep. 2020 to Dec. 2020. The screening criteria were as follows: (1) diagnosed as ACS [13]; (2) diagnosed as normal serum creatinine [11]; (3) agreed to the study protocol. The exclusion criteria included severe cardiomyopathy, coronary artery spasm angina, valvular heart disease, systemic inflammatory disease, autoimmune disorder, malignant neoplasms, hepatic and renal dysfunction, vitamin treatment or vitamin supplementation, and inability to act in accordance with the protocol. Finally, one hundred and sixty-five ACS patients were included in the present study. Informed written consent was obtained from each patient and the study protocol was approved by local ethical committee according to the Declaration of Helsinki.

### Demographic and anthropometric measurement

Demographic and clinical features of all subjects including age, sex, body mass index (BMI), systolic blood pressure (SBP), diastolic blood pressure (DBP), as well as history of smoking, hypertension, and diabetes were recorded. BMI was calculated as weight in kilograms divided by the square of the height in meters (kg/m^2^). Blood pressure in sitting position was remeasured twice in 5-min interval and averaged. Smoking status was confirmed by the medical history. Hypertension was defined as SBP ≥ 140 mmHg and/or DBP ≥ 90 mmHg, or having a history of hypertension or current treatment with antihypertensive medications. The diagnosis of diabetes was based on the World Health Organization criteria [14] or having a history of diabetes or current treatment with antidiabetic medications.

### Biochemical measurement

All blood samples were collected with anticoagulant tubes in the morning after an overnight (12 h) fast during hospital stay. All biochemical analyses were conducted at the accredited hospital laboratories on the day of blood collection. Serum homocysteine levels were analyzed via the enzymatic cycling method. Serum levels of fasting glucose, total cholesterol (TC), low-density lipoprotein cholesterol (LDL-C), high-density lipoprotein cholesterol (HDL-C), total triglyceride (TG), lipoprotein (LPa), apolipoprotein A1 (Apo A1), apolipoprotein B (Apo B), creatinine, cystatin C, blood urea nitrogen (BUN), uric acid (UA), and albumin were determined with standard methods on an automatic biochemistry analyzer (Roche Cobas-c702 modular analyzer). Levels of hemoglobin, platelets and white blood cell (WBC) were determined using a Sysmex XN-9000 analyzer.

### Coronary angiography

Selective coronary angiographies were performed with the standard Judkin’s technique by filming of multiple views of each blood vessel. Coronary angiograms were assessed by two experienced interventional physicians who knew nothing about the clinical characteristics of the participants. The diagnostic criterion of coronary artery disease was the individuals with at least one obvious stenosis (> 50%) of the lumen diameter in any of the major coronary arteries, including the left main coronary artery, left anterior descending artery, left circumflex coronary artery and right coronary artery, or main branches of the vascular system [15].

### Statistical analysis

Statistical analyses were performed using IBM SPSS (Statistical Package for the Social Sciences) for windows 23.0 statistical software package. Values of *P* < 0.05 were considered to be statistically significant. The standard distribution of the continuous variables was evaluated using the Kolmogorov–Smirnov test. Continuous variables with a standard distribution were shown as mean ± SD and those with a non-Gaussian distribution were shown as median (25th–75th percentile). Student’s *t* test was used for the comparison of normally distributed continuous numerical variables while the Mann–Whitney *U* test was used for non-normally distributed numerical variables. The categorical variable was expressed as number of cases (*n*) and percentage (%), and categorical data between the groups were compared by chi-square test. For variables with a skewed deviation, logarithmic transformations were employed. Correlational analysis was performed using bivariate Pearson’s correlation coefficients or Spearman’s rank correlation. It could be considered as excellent correlation, good correlation, average correlation or absence of correlation if correlation values were 0.7–1.0, 0.4–0.7, 0.2–0.4, and 0–0.2, respectively. Multivariate linear regression analyses with a stepwise selection method were employed to assess correlations of homocysteine (log_10_) with clinical and metabolic parameters.

## Results

A total of 165 patients were included in this study. The mean age of the subjects was 62.7 ± 11.0 years and 105 (64%) of the participants were men. The anthropometric and laboratory characteristics of the enrolled patients classified by gender are presented in Table 1.

**Table 1:** Anthropometric and laboratory characteristics of study population

Variables	Male (*N* = 105)	Female (*N* = 60)	*P* value
Age (year)	60.62 ± 11.70	66.25 ± 8.55	0.001
BMI (kg/m^2^)	25.99 ± 3.10	25.34 ± 3.78	0.237
SBP (mmHg)	140.12 ± 19.13	142.57 ± 18.10	0.422
DBP (mmHg)	86.00 ± 10.70	84.13 ± 11.76	0.300
Smoking (%)	55(52.4)	5(8.3)	0.0001
Hypertension (%)	79(75.2)	37(61.7)	0.066
Diabetes (%)	25(23.8)	22(36.7)	0.078
Fasting glucose (mmol/L)	5.99 ± 1.94	6.07 ± 1.75	0.785
TC (mmol/L)	4.04 ± 1.00	4.69 ± 1.30	0.001
LDL-C (mmol/L)	2.37 ± 0.86	2.77 ± 1.09	0.018
HDL-C (mmol/L)	1.03 ± 0.24	1.14 ± 0.27	0.006
TG (mmol/L)	1.18(0.78–1.73)	1.35(0.92–1.75)	0.167
LPa (nmol/L)	32.30(14.05–80.40)	50.50(15.25–138.45)	0.150
Apo A1 (g/L)	1.20 ± 0.19	1.32 ± 0.19	0.0001
Apo B (g/L)	0.95 ± 0.28	1.08 ± 0.36	0.012
Hemoglobin (g/L)	144.71 ± 15.14	126.77 ± 12.35	0.0001
WBC (10^9^/L)	6.95 ± 1.94	6.29 ± 1.80	0.032
Platelets (10^9^/L)	222.85 ± 62.03	215.88 ± 51.39	0.462
Creatinine (μmol/L)	77.57 ± 12.54	61.47 ± 12.28	0.0001
Cystatin C (mg/L)	0.98(0.89–1.10)	0.97(0.86–1.12)	0.862
BUN (mmol/L)	5.20(4.27–6.20)	4.60(3.91–6.09)	0.300
UA (μmol/L)	332.12 ± 95.24	265.08 ± 98.36	0.0001
Albumin (g/L)	42.29 ± 4.11	42.32 ± 5.08	0.970
Homocysteine (μmol/L)	14.80(12.40–19.10)	12.55(10.15–13.55)	0.0001

Continuous variables are shown as the mean ± SD or median (25th–75th percentiles). Categorical variable is expressed as number of cases (n) and percentage (%). The abbreviations of the variables: *BMI* body mass index, *SBP* systolic blood pressure, *DBP* diastolic blood pressure, *TC* total cholesterol, *LDL-C* low-density lipoprotein cholesterol, *HDL-C* high-density lipoprotein cholesterol, *TG* triglyceride, *LPa* lipoprotein a, *Apo A1* apolipoprotein A1, *Apo B* apolipoprotein B, *WBC* white blood cell, *BUN* blood urea nitrogen, *UA* uric acid

As shown in Table 1, male participants were more commonly had a history of smoking and the average age was younger in male compared with female subjects. However, there were no significant differences between the two groups in terms of BMI, SBP, DBP, hypertension and diabetes. Males were found to have significantly higher plasma homocysteine concentrations than females. The circulating levels of hemoglobin, WBC, creatinine and UA were significantly higher while TC, LDL-C, HDL-C, ApoA1 and ApoB were significantly lower in male patients compared with female subjects. However, the differences in fasting glucose, TG, LPa, platelets, cystatin C, BUN and albumin between the groups were not significant.

The correlations of homocysteine (log_10_) with clinical variables in males and females are shown in Table 2. In males, a significant positive correlation was found between serum homocysteine (log_10_) and hypertension (*r* = 0.569, good correlation, *P* < 0.001), creatinine (*r* = 0.367, average correlation, *P* < 0.001) and cystatin C (log_10_) (*r* = 0.333, average correlation, *P* = 0.001). In females, serum homocysteine (log_10_) was positively correlated with age (*r* = 0.307, average correlation, *P* = 0.107), hypertension (*r* = 0.456, good correlation, *P* < 0.001), creatinine (*r* = 0.341, average correlation, *P* = 0.008), cystatin C (log_10_) (*r* = 0.429, good correlation, *P* = 0.001) and UA (*r* = 0.569, good correlation, *P* < 0.001) whereas was negatively associated with LDL-C (*r* =  − 0.298, average correlation, *P* = 0.021) and ApoB (*r* =  − 0.273, average correlation, *P* = 0.033). Parameters up to statistical significance in males or females, for example age, hypertension, LDL-C, ApoB, creatinine, cystatin C (log_10_) and UA, were incorporated into the stepwise linear regression models to determine which parameters were independently associated with homocysteine.

**Table 2:** Correlation coefficients of serum homocysteine with other clinical variables

Variables	Homocysteine (log_10_)			
	Male		Female	
	Correlation coefficients	*P* value	Correlation coefficients	*P* value
Age	0.152	0.121	0.307	0.017
BMI	− 0.024	0.806	0.214	0.101
SBP	0.180	0.065	0.126	0.339
DBP	0.171	0.082	− 0.013	0.922
Smoking	− 0.034	0.728	− 0.192	0.143
Hypertension	0.569	< 0.001	0.456	< 0.001
Diabetes	0.068	0.493	0.018	0.889
Fasting glucose	− 0.090	0.361	− 0.002	0.989
TC	0.082	0.408	− 0.225	0.084
LDL-C	0.104	0.289	− 0.298	0.021
HDL-C	− 0.074	0.455	− 0.120	0.361
TG (log_10_)	0.034	0.730	0.034	0.797
LPa (log_10_)	− 0.182	0.063	− 0.178	0.173
Apo A1	− 0.125	0.203	− 0.066	0.615
Apo B	0.086	0.381	− 0.273	0.033
Hemoglobin	− 0.047	0.636	− 0.129	0.328
WBC	0.033	0.738	0.043	0.744
Platelets	0.008	0.938	0.023	0.860
Creatinine	0.367	< 0.001	0.341	0.008
Cystatin C (log_10_)	0.333	0.001	0.429	0.001
BUN (log_10_)	0.139	0.158	− 0.016	0.902
UA	0.192	0.050	0.295	0.022
Albumin	− 0.068	0.488	0.054	0.684

The abbreviations of the variables: *BMI* body mass index, *SBP* systolic blood pressure, *DBP* diastolic blood pressure, *TC* total cholesterol, *LDL-C* low-density lipoprotein cholesterol, *HDL-C* high-density lipoprotein cholesterol, *TG* triglyceride, *LPa* lipoprotein a, *Apo A1* apolipoprotein A1, *Apo B* apolipoprotein B, *WBC* white blood cell, *BUN* blood urea nitrogen, *UA* uric acid

As shown in Table 3, multiple stepwise regression analysis showed that after adjusting for the confounders, hypertension (*P* < 0.001) and creatinine (*P* = 0.031) were independently related to homocysteine (log_10_) in men. This model had an adjusted *R*-squared value of 0.299. Of note is that hypertension alone determined most of the variability of homocysteine levels in males in the full model including hypertension and creatinine as independent regressors.

**Table 3:** Multivariate regression analysis of homocysteine (log_10_) with clinical and metabolic parameters in males

Variables	Full model*		
	β ± SE	Standardized β	*P* value
Constant	0.901 ± 0.076		< 0.001
Hypertension	0.148 ± 0.030	0.440	< 0.001
Creatinine	0.002 ± 0.001	0.196	0.031
			Model *R*^2^
Full model*			0.299
Hypertension only-model			0.266
Creatinine only model			0.135

The table above shows significant (*P* < 0.05) independent regressors of serum homocysteine (log_10_) determined from stepwise multivariable linear regression modeling. *The full model includes hypertension and creatinine but not age, LDL-C, apoB, UA or cystatin C (log_10_) as independent regressors. The abbreviations of the variables: LDL-C, low-density lipoprotein cholesterol; Apo B, apolipoprotein B; UA, uric acid

As shown in Table 4, multiple stepwise regression analysis showed that after adjusting for confounding factors, cystatin C (log_10_) (*P* = 0.004) and hypertension (*P* = 0.005) were independently related to homocysteine (log_10_) in women. This model had an adjusted *R*-squared value of 0.290. It is noteworthy that cystatin C alone determined over half of the variability of homocysteine levels in females in the full model including cystatin C and hypertension as independent regressors.

**Table 4:** Multivariate regression analysis of homocysteine (log_10_) with clinical and metabolic parameters in females

Variables	Full model*		
	β ± SE	Standardized β	*P* value
Constant	1.039 ± 0.020		< 0.001
Cystatin C (log_10_)	0.464 ± 0.154	0.346	0.004
Hypertension	0.076 ± 0.026	0.336	0.005
			Model *R*^2^
Full model*			0.290
Cystatin C (log_10_) only model			0.184
Hypertension only model			0.177

This table shows significant (*P* < 0.05) independent regressors of fasting serum homocysteine determined from stepwise multivariable linear regression modeling. *The full model includes cystatin C (log_10_) and hypertension but not age, LDL-C, apoB, UA or creatinine as independent regressors. The abbreviations of the variables: *LDL-C* low-density lipoprotein cholesterol, *Apo B* apolipoprotein B, *UA* uric acid

## Discussion

The main determinants of fasting plasma homocysteine levels were hypertension and creatinine in men while cystatin C and hypertension in women in ACS patients with normal renal function. Our data supported the notion that there are gender-related differences in the determinants of plasma homocysteine levels in ACS subjects with normal kidney function.

The sex-related differences of serum levels of homocysteine had been noted by previous studies. Rauh et al. [16] reported that adult men had significantly higher homocysteine levels than women while there are no significant differences between boys and girls. The study by Chou and colleagues demonstrated that male subjects had higher homocysteine levels than female participants and the mean levels of plasma homocysteine were significantly higher in occluded coronary artery disease or in maintenance hemodialysis than in age-matched normal subjects [17]. Moreover, fasting plasma homocysteine levels were significantly elevated in adult men compared to women among coronary artery disease subjects including ACS patients [5, 18]. In the present study, we found that the gender-associated difference also existed in ACS patients with normal renal function, with higher plasma concentration of homocysteine in adult men. In line with previous works [19, 20], our results indicated that men had higher smoking rate, creatinine, and UA compared with women, whereas women had higher age than men. In the present study, we also found that men had lower TC, LDL-C, HDL-C, ApoA1 and ApoB compared to women, which is consistent with previous studies [20–23]. The serum levels of WBC and hemoglobin were higher in male participants than female subjects in our study, which is in agreement with previous report [24].

The raised serum homocysteine levels in men might be associated with the fact that men have more muscle mass than women and most homocysteine is formed along with the formation of creatine-creatinine [8]. On the other hand, it seems that females are better adapted to avoid high homocysteine through the transsulfuration pathway than males [22]. Other possible explanation might be the differences in hormone and vitamin status [25] though this issue is not concluded [6].

Previous studies have investigated gender-associated differences in risk factors for elevated homocysteine concentrations in healthy subjects; however, the results were inconsistent. For example, Xu and colleagues reported that the factors associated with homocysteine concentration in males were smoking status, estimated glomerular filtration rate, BUN, DBP, free triiodothyronine, serum potassium and cystatin C while in females, the factors were cystatin C, albumin, free thyroxine, age, free triiodothyronine and serum potassium [19]. The study by Zhao et al. [23] demonstrated that age, BMI and TG were independent risk factors for homocysteine levels specifically in women though common correlational factors (aspartate aminotransferase, creatinine, UA, LDL-C and HDL-C) existed in both genders. In this study, we found that the factors associated with homocysteine levels in males were hypertension, creatinine and cystatin C while in females, the factors were age, hypertension, LDL-C, Apo B, creatinine, cystatin C and UA. The divergent results above may partly be explained by the different inclusion criteria and the different sample size. In present study, we further discovered that the main determinant of fasting serum homocysteine levels in men and in women was hypertension and renal function parameters, respectively, in ACS patients with normal creatinine levels. Of note is that cystatin C was the strongest determinant of serum homocysteine in women in ACS participants with normal kidney function. It is in line with the study by Norlund et al. [8], which showed that plasma cystatin C exhibited the highest predictive value for plasma homocysteine concentration in healthy women. In our study, plasma creatinine, but not cystatin C, was the correlate of serum homocysteine in men in ACS participants with normal renal function. It is inconsistent with the study by Norlund et al. [8], which indicated that plasma creatinine showed a lower explanatory power than cystatin C and age in healthy men. It might be related to the influence of disease status and the comorbidities in ACS subjects, which is different from the condition of health person. Additional studies are warranted to explore gender-associated differences in renal function parameters for elevated homocysteine concentrations in ACS patients with normal kidney function.

As mentioned in Introduction section, renal function is an important determinant of serum homocysteine levels. There is a positive association between serum homocysteine and renal function parameters in renal failure patients [9] or in normal renal function subjects [10]. Cystatin C is not related to the muscle mass and formation of creatinine. Thus, cystatin C had been considered a better marker of glomerular filtration rate than serum creatinine. Cystatin C was positively correlated with homocysteine levels in coronary artery disease patients without chronic kidney disease [26]. Bostom et al. [11] reported that cystatin C, but not serum creatinine, could independently predict serum total homocysteine among stable coronary artery disease patients with normal renal function. In addition, the study by TURGAN et al. demonstrated that serum creatinine was positively associated with serum homocysteine in ACS patients with normal renal function [27]. However, it seemed that the impairment of kidney function had only a minor influence on serum homocysteine levels in subjects with ACS [12]. The discrepancies above might be explained by the small sample size in the studies. With larger sample size, we discovered that subclinical renal dysfunction was an important determinant of serum homocysteine levels in cardiovascular disease. This view had been supported by previous studies [7, 8, 11, 28].

In the present study, the main determinants of fasting plasma homocysteine levels, in addition to renal function parameters, were hypertension in male and female ACS patients with normal creatinine levels. It is worthwhile to note that hypertension showed a higher explanatory power than renal function parameters in male ACS participants with normal renal function. In China, hyperhomocysteinemia is common in subjects with hypertension [29]. In fact, many clinical studies have suggested that there is a close relation between homocysteine and hypertension, the detailed mechanisms of which are not fully elucidated [30–32]. The data from China demonstrated that homocysteine levels were independent risk factors that are positively associated with increased serum creatinine and BUN levels in male patients with hypertension [33]. Of note is that any relationship between homocysteine levels and blood pressure might be confounded by renal function [30, 31]. Thus, the subclinical renal dysfunction in hypertension subjects may play an important part in the relationship between homocysteine and hypertension. In addition, homocysteine and hypertension might influence each other via other metabolic abnormalities, for example insulin resistance [30, 31]. Nonetheless, our study emphasized the importance of controlling hypertension and hyperhomocysteinemia together in ACS patients, even with normal renal function.

This study has several limitations. First, this study was a cross-sectional study and lack of long-term follow-up data. Second, there is limited number of participants in the study due to the limited funding, which might influence its statistical power. Third, all the participants in this study were inpatients and underwent coronary angiography, which might cause selection bias. Fourth, this study was lack of information about dietary factors, vitamin B12 and folate status, and genetic factors related to homocysteine metabolism since these variables might influence homocysteine levels.

In conclusion, hypertension and cystatin C could explain most of the sex differences in serum homocysteine levels in ACS subjects with normal serum creatinine. Our study indicated the importance of making different strategies in males and females to handle hyperhomocysteinemia effectively in ACS subjects without renal dysfunction. Future studies with larger groups of subjects are needed to explore the sex-related relationship of hypertension, cystatin C and hyperhomocysteinemia in ACS patients with normal renal function.

## Data Availability

Considering the privacy of participants, if readers have a need to obtain data related to this article, they could contact the corresponding author.

## Abbreviations

ACS: Acute coronary syndrome
BMI: Body mass index
SBP: Systolic blood pressure
DBP: Diastolic blood pressure
TC: Total cholesterol
LDL-C: Low-density lipoprotein cholesterol
HDL-C: High-density lipoprotein cholesterol
TG: Triglycerides
LPa: Lipoprotein
Apo A1: Apolipoprotein A1
Apo B: Apolipoprotein B
WBC: White blood cell
BUN: Blood urea nitrogen
UA: Uric acid
